# Iconic Therapy for the reduction of borderline personality disorder symptoms among suicidal youth: a preliminary study

**DOI:** 10.1186/s12888-022-03862-x

**Published:** 2022-03-29

**Authors:** Silvia Hurtado-Santiago, José Guzmán-Parra, Fermín Mayoral, Rosa M. Bersabé

**Affiliations:** 1 San Juan de Dios Psychiatric Centre, Málaga, Spain; 2 University Regional Hospital of Málaga. Mental Health Unit. Biomedical Research Institute of Málaga (IBIMA, Málaga, Spain; 3 Psychobiology and Methodology of the Behavioral Sciences Department, University of Málaga, Málaga, Spain

**Keywords:** Iconic therapy, Borderline personality disorder, Psychological therapy, Suicide, Clinical trial

## Abstract

**Background:**

Iconic therapy (IT) is a new therapy that uses images to teach skills with the aim of improving the symptoms of borderline personality disorder. Preliminary results are promising, and there is indication that IT may be effective. The purpose of this preliminary study was to test the effectiveness of IT compared to a psychological supportive intervention (SI).

**Methods:**

The study was carried out at the University Regional Hospital of Malaga. Young patients (*N* = 40; 15–30 years) with suicidal or parasuicidal behavior and borderline personality traits were randomized into IT (*N* = 20) or SI (*N* = 20). The main outcome variable was a change in the symptoms of borderline personality disorder (BSL-23) at the end of treatment. The secondary outcome variables were suicidal ideation and behavior, self-harm, the need for medication, the number of visits to mental health professionals, maladaptive behavior, satisfaction with therapy and perceived improvement, both at the end of the intensive treatment and at the 12-month follow-up.

**Results:**

As expected, the two therapies produced a reduction in BPD symptoms at 10 weeks post-treatment and at the 12-month follow-up. Contrary to expectation, there were no statistically significant differences in the effectiveness of the two therapies (*p* > 0.05). However, at the 12-month follow-up, the effect sizes for the difference between the effectiveness of the two therapy groups on BSL-23 scores (*d* = 0.33) and on maladjustment to daily life (*d* = 0.39) was found to exceed the commonly used convention for a small effect (*d* = 0.20). Besides, participants in the IT group showed greater satisfaction with therapy than those who received SI. The mean difference between groups was statistically significant after the 10-week treatment period (*p* < .01), with a large effect size (*d* = 1.11). Nevertheless, this difference was not maintained at the 12-month follow-up (*p* > .05), although the effect size for this analysis (*d* = 0.34) was found to exceed a small effect.

**Conclusions:**

This preliminary study did not find a statistically significant difference in the effectiveness of the two therapies, probably due to the small sample of participants, but there are some indicators (effect sizes) suggesting that perhaps IT may be superior for reducing BPD symptoms and maladjustment in daily life. Future studies with larger samples and comparisons with established treatments for borderline personality disorder are necessary to confirm that IT effects are significant and persistent in the long term.

**Trial registration:**

ClinicalTrials.gov identifier: NCT03011190. First posted 05/01/2017. Last update posted 15/05/2018.

## Background

Borderline personality disorder (BPD) is characterized by a pattern of instability in the regulation of emotions, self-image, behavior control and interpersonal relationships [1]. BPD is especially prevalent in young individuals, with a peak prevalence in early adulthood [2]. At this stage of life, the interaction of temperamental, environmental and genetic factors with early traumatic experience and a dysfunctional familial environment can promote the onset of BPD symptoms [3]. In young populations, the symptoms of BPD are associated with an increased risk of suicide [4]. Suicidal ideation usually moves adolescents to seek professional help [5]. Preventive psychological treatment in the early stages of the disorder may be the best long-term strategy, as new studies indicate high flexibility in BPD traits at this stage and adequate response rates to the treatments studied [6, 7]. However, suicide attempts are particularly difficult to keep under control through psychotherapy in adolescents, according to a recent metanalysis [8].

Among the evidence-based treatments for BPD, according to clinical practice guidelines, are psychological treatments [9]. Those psychological treatments showing more evidence of effectiveness are dialectic behavioral therapy, mentalization-based therapy and transference-focused therapy [10]. These therapies are very intensive and require the use of many therapeutic sessions over long periods of time, which is often a major impediment for patients experiencing mood instability in terms of completing the treatment. In addition, most of the evidence on these therapies comes from studies conducted at centers of excellence, so their generalizability is not well-established [9]. However, there is no evidence of the superiority of prolonged treatments compared to brief treatments for BPD [11, 12].

Recent research also shows that BPD is associated with cognitive deficits in executive function [13] and attention [14]. These alterations can hinder the brief traditional psychotherapeutic approach and, therefore, any kind of extra support to increase their effectiveness may be helpful. Visual aids could be an alternative, as images can improve learning [15] and also promote the creation of positive mental representations, which have been shown to have a causal role in mood and can even improve depressive symptoms [16].

Iconic therapy (IT) is an integrative therapy that uses images to teach attitudes and skills for improving the symptoms of BPD. Its major characteristic is a brief, highly structured program to help those affected by BPD acknowledge their emotions and improve their ability to function. The program includes both an explicative model of unstable behaviors and a therapeutic model. The explicative model addresses the origins and mechanisms of perpetuation of unstable relationships, moods, thinking and behaviors, so that BPD patients feel validated and increase their cognitive insight. To do so, the therapist, in a classroom setting, similar to a psychoeducational seminar or workshop, asks to the participants about their usual frustration reactions (i.e. anger, attacks, apathy, withdrawal, crying, self-harming) and derived consequences (new problems and more frustration). Their responses are written on a blackboard and reformulated into five categories (self-aggression, hetero-aggression, escape, idealization or manipulation). That information is used to interactively organize and draw the IT explicative model, consisting of a negative vicious circle that usually makes participants recognize their problem and feel curiosity to know more. Interconnected with arrows in a flowchart, the therapeutic model orders and adapts interventional principles of existing current treatments to increase the chances of therapeutic success, such as *key phrase* for initial attitude shift [17], *cooling down* or distracting activities to be able to accept the problem and have perspective over it [18, 19], *problem solving for those situations in which a decision must be taken* [20], *social skills or necessary assertive communication* to reach agreements [21] and some author contributions to *learning from mistakes* by analyzing and making little changes to succeed or *persistence towards a vital goal* and to not abandon it when frustrated [22]. This structure helps patients head from unstable behaviors to more adaptive ones in a kind of “visual step by step map”. Simultaneously, IT works on BPD vulnerability factors: dichotomy [23], rumination [24], low self-esteem [25, 26], wrong beliefs [25], external control locus [27] or codependence [26]. At the end of each and every group session, the IT materials are handed out so participants can keep them as a resource during difficult periods”.

Iconic Therapy’s denomination is due to the symbolic representation of given therapeutic principles (acceptance, problem solving, resilience, self-direction or empathy) in a total of 35 images (icons). Images include generic objects of our environment (such as a table, a sailboat or a tower of books) whose emotional valence is neutral. These icons, together with the flowchart (or map) of the program, simplify the explanation of a wide variety of complex social situations in which the capacity of BPD patients to learn is adversely affected [28]. In summary, IT provides a much-needed full panoramic vision of BPD that brings awareness (insight) and intention (control) for affected patients to cope with everyday tasks. Iconic Therapy’s good acceptance among adolescents may be precisely related to their natural use of images and metaphors in daily life. Guidelines for IT have been prepared and are currently pending publication. It contains examples and a number of therapists’ considerations that may easily be translated and exported to many other languages and cultures. The program is divided into four modules, focusing on basic coping skills, interpersonal relationships, self-esteem and identity and, finally, self-direction. In clinical practice, this therapy has been developed as an initial intensive 10–12-week phase of weekly group sessions and variable individual sessions. The second phase of treatment consists of a 12-month follow-up with individual support sessions. Some preliminary studies have shown promising results, but with the limitation of small samples [29, 30]. So far, no controlled clinical trial on IT has been carried out.

The objective of the study was to analyze the effectiveness of IT for reducing symptomatology in young individuals with BPD traits and suicidal behavior in comparison with psychological supportive intervention (SI). The primary outcome of the study was a decrease in the severity of the BPD symptoms at the end of the 10-week intensive treatment and at the 12-month follow-up.

## Methods and materials

### Procedure

The study was a two-armed parallel randomized controlled trial (RCT). The Provincial Ethics and Research Committee of Malaga approved and registered the study (shs-ico-2015-01). Informed consent to participate was obtained from all participants and parental consent was obtained for participants under the age of 18 years. The sample size calculation was based on our primary outcome measure, i.e. BPD symptom severity at 12 months. A sample size of 26 participants per group was required to detect a post-treatment effect size of 0.70 (Cohen’s d) between both groups with a power of 0.80 in a one-tailed test, and with a 95% confidence level. The total sample size was then determined at 52 participants, but only 40 individuals could finally be recruited. More details about the trial have been published previously in the study protocol [31]. In short, the study was conducted at the University Regional Hospital of Malaga during January 2016 and July 2017. Patients were informed of the study by psychologists and psychiatrists working in the public community care system and if they were interested in participating a study researcher was notified. If the participants met the inclusion criteria, they were randomized in a 1:1 ratio by the principal investigator into two interventions: IT or SI (which does not use images during the therapeutic process). In the two comparison groups, participants followed their usual treatment within the public health system. All the therapists were volunteers with a Master’s degree in psychology. Four therapists and four co-therapists administered the two therapy groups, forming a total of four groups (two for each intervention). It was inherently not possible to blind psychologists delivering the IT intervention since they had been specifically trained for 10 months. The goal of the psychologists conducting the control group was to obtain good clinical results through therapeutic intervention. Also, two group sessions were recorded to assess the fidelity of the therapy. Supervision and feedback were given to the therapist and co-therapist by the principal investigator during the study for both therapy groups. It was considered that the participants had completed treatment if they attended at least 80% of the sessions.

This study was approved by the Ethics and Research Committee of the Regional University Hospital of Málaga. The study design and performance complied with the Declaration of Helsinki.

### Participants

The planned sample size was not recruited due to a progressive decrease in the flow of derivations from the public community care system. Regarding the inclusion criteria, a typo related to the age range was detected in the study protocol. We accidentally mentioned 15 to 25 years old instead of 15 to 30 years as initially planned. We reported the mistake to the Ethics Committee as soon as it was detected, prior to the beginning of the treatments. The sample of this study was composed of 40 participants: *N* = 20 in the IT group and *N* = 20 in the control group. The inclusion criteria were 15–30 years old, presence of suicidal ideation (two or more questions about suicidal ideation affirmatively reported in the Columbia Suicide History Form), borderline personality symptoms (score of ≥38 out of 84 on the Exploratory Questionnaire of Personality-III-BPD) and sufficiently proficient in Spanish to conduct the treatment. The exclusion criteria were antisocial personality disorder, substance/alcohol abuse or dependence and not enough motivation to participate in treatment (score of ≥35 out of 60 points on the Credibility/Expectancy Questionnaire). Recruitment lasted 2 months (November and December’15). Clinical interventions took place between January and June 2016. The study ended in July 2017. The sociodemographic characteristics of the participants are shown in Table 1.

**Table 1:** Sociodemographic characteristics of the randomized sample at baseline and attrition to treatment

*Variable*	Total *N* = 40		Support Therapy *N* = 20		Iconic Therapy *N* = 20		*χ*^2^ / *t*	*p*
Age (15–29), M (SD)	20.53	(4.32)	19.95	(4.37)	21.10	(4.30)	−0.83	.407
Gender (*Female*) (%)	33	(82.5)	15	(75.0)	18	(90.0)	1.55	.405
Civil Status (*Single*) (%)	40	(100)	20	(100)	20	(100.0)		
Parent’s nationality (Both Spanish) (%)	30	(75.5)	14	(70.0)	16	(80.0)	0.53	.465
Cohabitation with family of origin (%)	35	(87.5)	17	(85.0)	18	(90.0)	0.30	.633
Educational level (%)							4.46	.107
*Primary*	20	(50.0)	11	(55.0)	9	(45.5)		
*Secondary*	13	(32.5)	8	(40.0)	5	(25.0)		
*Universitary*	7	(17.5)	1	(5.0)	6	(30.0)		
Laboral Status (%)							0.46	.792
*Unemployed*	14	(35.0)	6	(30)	8	(40)		
*Partial Time Job*	4	(10.0)	2	(10)	2	(10)		
*Full Time Job*	22	(5.5)	12	(60)	10	(50)		
Complete follow-up (%)	32	(80.0)	15	(75)	17	(85)	0.62	.693
Complete the treatment							2.56	.110
*(Number of group sessions* ≥ 8) (%)	23	(59.0)	9	(45.0)	14	(70.0)		
Number of individual sessions, M (SD)	3.15	(3.39)	2.21	(2.37)	4.05	(3.99)	−1.73	.091

### Instruments

The primary outcome measure was the severity of BPD assessed with the *Borderline Symptom List* (BSL-23) [32]. The secondary outcome measures were: suicidal ideation with the *Columbia-Suicide Severity Rating Scale* [33]; suicide attempts; non-suicidal self-injury assessed following the criteria from DSM-V [34] (suicide attempts and non-suicidal self-injury were evaluated for 12 months before baseline, during treatment and at follow-up); and maladjustment to daily life with the *Maladjustment Scale* [35]. Because of the underpowered sample size to conduct the economic analysis, it was decided not to use the *Client Service Receipt Inventory* [36] in the study protocol and only collect information on the medication and visits to mental health professionals. After the treatment was evaluated, satisfaction with treatment was assessed with an adaptation of the *Credibility/Expectancy Questionnaire* [37] and perceived global improvement from participants and family/friends (seven-point Likert scale). The following sociodemographic variables were assessed at baseline: gender, age, marital status, parent’s nationality, cohabitation, educational level and occupational status.

### Interventions

#### Iconic therapy

Therapy consisted of a 10-session program structured into four modules: motivation and coping skills, intimacy/empathy, identity and self-direction. Each module had two or three weekly group sessions 90 min in duration. In the first 30 min, practical use of the last session’s icons was discussed and then new icons were presented. In the second part of the program, supportive individual face-to-face sessions were conducted by the principal investigator. Additionally, three more group sessions were conducted after treatment and at 6- and 12-month follow-ups. The objective of these sessions was to minimize drop-outs and try to meet the specific needs of the participants. A therapist and co-therapist conducted the group sessions. The IT program was delivered by therapists trained in IT specifically for this study and group sessions were supervised from behind a unidirectional window by the principal investigator. The four therapists who delivered this therapy had undertaken 10 months of training (10 sessions).

#### Psychological supportive intervention

The SI treatment consisted of a 10-session program of 60 min per week. The sessions were 30 min shorter than the IT sessions because the verbal nature of the intervention does not require as much practical clarification by the therapists and long sessions could affect adherence. This therapy had a psychoeducational nature, with modules focused on the improvement of social relationships and skills, assertiveness, self-esteem, impulse control and learning relaxation techniques and mindfulness. As in IT, the second part of the program consisted of three group sessions and a variable number of individual face-to-face sessions conducted by the principal investigator. More information about the content of both IT and SI is given in the previously published protocol [31].

### Statistical analysis

An intention-to-treat analysis was carried out on all the participants, irrespective of whether they were completers. Demographic characteristics were summarized with descriptive statistics and differences between treatment groups were examined using the chi square test for categorical variables and *t*-tests for continuous variables if normality was fulfilled (if not, the Mann-Whitney U test was used). Also, for suicide attempts and non-suicidal self-injury, McNemar’s test was conducted to analyze the change between the previous year of baseline and at follow-up for these variables.

Analyses for primary and secondary outcomes were carried out using generalized estimating equation (GEE) models. In brief, GEE models are an extension of generalized linear models for repeated measures or correlated observations. In addition, GEE models can also deal with data that have missing values, as long as the missing value is completely random. This means that analyses are computed with all participants (in this study, *N* = 40), even if some of them have a missing value. Parameter estimation can still yield robust results.

Intervention conditions (IT vs. SI) were included for the between-subjects factor and time (baseline, after treatment and at the 12-month follow-up) as the repeated measure. The main effects and interactions between treatment and time were analyzed. A sensitivity analysis was carried out with the different correlation matrices (independent, AR [1], interchangeable, M-dependent and unstructured) and the model with the lower QIC (Quasilikehood under the Independence model Criterion) was selected. IBM SPSS (Version 22.0 for Windows) software was used and statistical significance was considered at *p* < .05. The Bonferroni correction was applied for post hoc multiple comparisons.

## Results

**Fig. 1:**
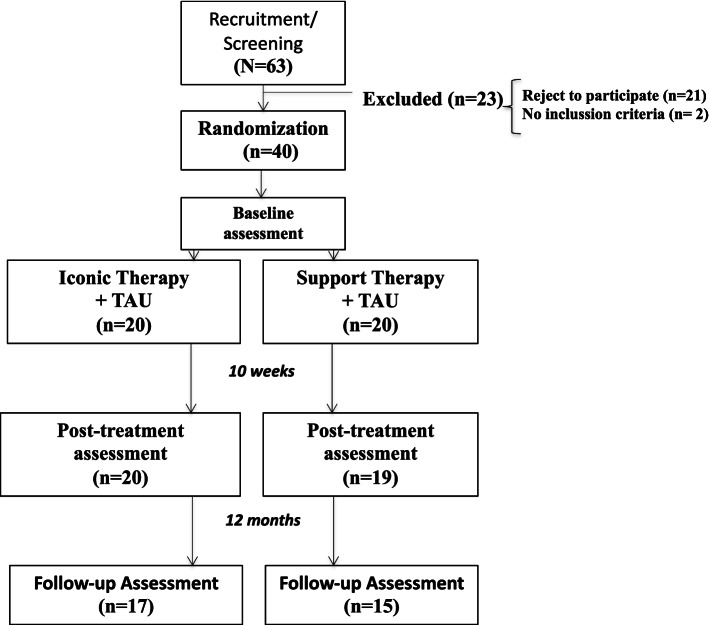
Flowchart

As shown in Table 1, there were no significant differences in the sociodemographic variables between the two groups at baseline. The information about recruitment is shown in a flow-chart diagram in Fig. 1. IT showed higher adherence compared to SI (70% vs. 45%), although the difference was not statistically significant (*p* = 0.110), as well as a lower drop-out rate at the last follow-up (15% vs. 25%), although these differences were also not significant (*p* = 0.429).

Table 2 shows the descriptive statistics of the four outcome variables that were assessed at three time points (baseline, post-treatment and follow-up): (1) BPD symptoms, (2) suicide ideation, (3) maladjustment to daily life and (4) psychopharmacology consumption. Analyses for these four variables were carried out using generalized estimating equation (GEE) models (see Table 3).

**Table 2:** Descriptive statistics of the outcome variables assessed at three moments, in each therapy group

		Baseline		Post- treatment (10 weeks)		Follow-up (12 months)	
Variable	Therapy	*M*	(*SD*)	*M*	(*SD*)	*M*	(*SD*)
BPD symptoms (BSL-23)	Support	52.25	(18.71)	42.63	(24.86)	37.13	(20.53)
	Iconic	51.40	(22.03)	40.10	(24.63)	28.06	(21.82)
	Cohen’s *d*			0.13^a^		0.33^b^	
Suicide Ideation (C-SSRS)	Support	4.00	(0.97)	2.68	(1.66)	1.33	(1.75)
	Iconic	3.55	(1.46)	2.00	(2.00)	1.00	(1.45)
	Cohen’s *d*			0.17^a^		0.04^b^	
Maladjustment to daily life (IG)	Support	23.30	(5.07)	19.21	(9.44)	16.27	(8.68)
	Iconic	20.60	(6.62)	15.10	(7.70)	9.82	(6.69)
	Cohen’s *d*			0.18^a^		0.39^b^	
Psychopharmacology consumption. *N* (%)	Support	14	70.0%	10	52.6%	8	53.3%
	Iconic	14	70.0%	11	55.0%	5	29.4%

Notes: ^a^ Cohen’s *d* for the mean difference between therapy groups on effectiveness at post-treatment;

^b^ Cohen’s *d* for the mean difference between therapy groups on effectiveness at follow-up

**Table 3:** Results of the Generalised Estimated Equations models (GEE)

Outcome variable	B	ES	95% CI	Wald	*P*
Symptomatology BPD (BSL-23)					
Therapy	0.850	6.302	−11.501/13.201	0.018	.893
Post-Treatment	−11.300	4.279	−19.687/−2.913	6.974	**.008**
Follow-up	−23.341	6.696	−36.467/-10.215	12.148	**.000**
Therapy*Post-Treatment	1.682	7.087	−12.208/15.572	0.056	.812
Therapy*Follow-up	8.225	8.090	−7.632/24.081	1.033	.309
Suicide ideation (C-SSRS)					
Therapy	0.450	0.384	−0.302/1.202	1.374	.241
Post-Treatment	−1.550	0.364	−2.263/− 0.837	18.149	**.000**
Follow-up	−2.550	0.499	−3.529/− 1.571	26.069	**.000**
Therapy*Post-Treatment	0.234	0.540	−0.824/1.292	0.188	.664
Therapy*Follow-up	−0.117	0.720	−1.527/1.294	0.026	.871
Maladjustment to daily life (IG)					
Therapy	2.700	1.819	−0.864/6.264	2.204	.138
Post-Treatment	−5.500	1.487	−8.415/− 2.585	13.672	**.000**
Follow-up	−10.776	1.819	−14.341/− 7.212	35.115	**.000**
Therapy*Post-Treatment	1.411	2.561	−3.609/6.430	0.303	.582
Therapy*Follow-up	3.743	3.225	−2.578/10.064	1.347	.246
Psychopharmacology consumption					
Therapy	1.009	0.742	−0.446/2.464	1.847	.174
Post-Treatment	−0.714	0.746	−2.176/0.749	0.915	.339
Follow-up	0.028	0.675	− 1.296/1.352	0.002	.967
Therapy*Post-Treatment	−0.362	0.960	−2.243/1.519	0.143	.706
Therapy*Follow-up	−1.104	.906	−2.280/0.671	1.486	.223

With respect to the primary outcome (BSL-23 score), the *Post-Treatment* main effect was statistically significant, which indicates that, as expected, both therapies showed significant reductions in the symptoms of BPD after 10 weeks of treatment. The *Follow-up* main effect was also statistically significant for BSL-23 scores. This means that the reduction in BPD symptoms was maintained at the 12-month follow-up, for both therapy groups. Therefore, both interventions produced a reduction in BPD symptoms at the end of the intensive treatment (10 weeks) and at the 12-month follow-up. However, contrary to the expected hypothesis, the results showed that there were no statistically significant differences between the effectiveness of the two interventions, as the interaction effects at the end of the treatment (Therapy*Post-Treatment) and at the follow-up (Therapy*Follow-up) were not statistically significant (see Table 3).

**Fig. 2:**
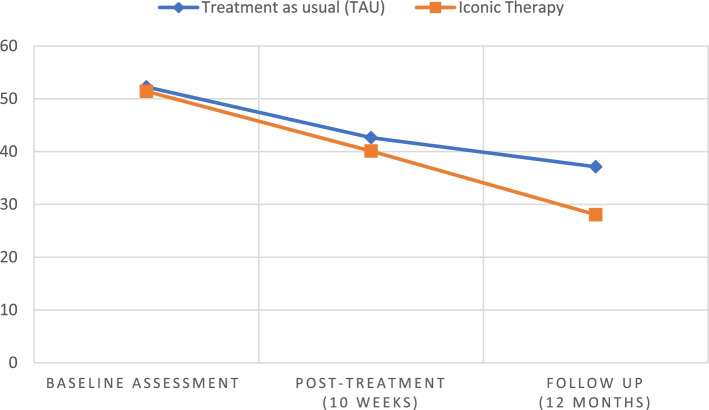
Mean BSL-23 scores in each therapy group, at the three assessment moments

Effect sizes were also computed to analyze the difference between the effectiveness of the two therapy types regarding the reduction in BPD symptoms (Table 2). The effectiveness of each therapy was measured at two timepoints: after 10 weeks of treatment (post-treatment *minus* baseline scores) and after 12 months of follow-up (follow-up *minus* baseline scores). After 10 weeks of treatment, the effect size was found to be below Cohen’s convention [38] for a small effect (*d* = 0.20). However, after 12 months of follow-up, the effect size for the difference between the effectiveness of the two therapy groups in BSL-23 mean scores was found to exceed a small effect. The results for the primary outcome in the three time periods can be seen in Fig. 2.

With respect to secondary outcomes assessed at three time points, the *Post-treatment* and *Follow-up* main effects were statistically significant for two variables: *suicidal ideation* and *maladjustment in daily life* (see Table 3). These results indicate that the two therapies showed an effect, both at the end of intensive treatment (10 weeks) and at the 12-month follow-up. On the contrary, the two therapies had no statistically significant effect on *psychopharmacology consumption* variable, as indicated by the *Post-Treatment* and *Follow-up* main effects.

Moreover, the results show that there was no significant difference between the effectiveness of the two interventions, as the interaction effect at the end of the treatment (Therapy*Post-Treatment) and the follow-up (Therapy*Follow-up) were not statistically significant for any of the three secondary outcome measures (Table 3). However, the effect size for *maladjustment to daily life* at follow-up was above a small effect (see Table 2).

Descriptive statistics for other secondary outcome variables, that were not assessed at baseline, are shown in Table 4. The results indicate that there were no statistically significant differences between the two therapy groups, either at post-treatment nor after follow-up, for any of the variables except for *satisfaction with therapy*. Participants in the IT group showed greater *satisfaction with therapy* (measured through the CEQ questionnaire) than those who received the support intervention. The mean difference between both groups was statistically significant after the 10-week treatment; the effect size for this analysis can be considered a large effect. However, at the 12-month follow-up, the mean difference between therapy groups did not reach statistical significance, although the effect size for this analysis was found to exceed a small effect.

**Table 4:** Differences in the outcome variables in the post-treatment and 12-month follow-up

		Post- treatment (10 weeks)			Follow-up (12 months)		
Variable	Therapy	*M*	(*SD*)	*χ*^2^/*t*	*M*	(*SD*)	*χ*^2^/*t*
Suicide Attempt (C-SSRS) *N* (%)	Support	9	47.3%	*χ*^2^=2.19	*2*	13.3%	*χ*^2^=0.521
	Iconic	5	25.5%		1	5.8%	
Non-Suicidal Self-injury (DSM-V) *N* (%)	Support	8	42.1%	*χ*^2^=0.03	6	40.0%	*χ*^2^=5.43^*^
	Iconic	9	45.0%		1	5.9%	
Visits to mental health professionals	Support	1.95	(2.99)	*t*=-0.11	4.82	(6.21)	*t*=0.632
	Iconic	1.85	(2.45)		3.80	(5.71)	
	Cohen’s *d*	0.04			0.17		
Satisfaction with therapy (CEQ)	Support	34.26	(9.92)	*t*=3.44^**^	44.13	(13.61)	*t*=0.947
	Iconic	43.40	(6.12)		47.88	(7.52)	
	Cohen’s *d*	1.11			0.34		
Subjective global improvement (Individual)	Support	3.16	(1.01)	*t*=-0.15	2.73	(1.83)	*t*=-0.709
	Iconic	3.10	(1.25)		2.35	(1.17)	
	Cohen’s *d*	0.05			0.25		
Subjective global improvement (Family)	Support	3.42	(0.84)	*t*=-1.12	2.40	(0.99)	*t*=1.921
	Iconic	3.05	(1.19)		3.12	(1.11)	
	Cohen’s *d*	0.36			0.68		

Notes: ^*^ *p* < 0.05 ^**^ *p* < 0.01 Bonferroni correction was applied

## Discussion

This clinical trial has shown that IT is feasible for application in a real clinical environment with therapists taught IT for the first time,and it was associated with greater satisfaction at the end of treatment than SI. This finding is consistent with the detected better adherence trend in IT compared with SI, although this difference only approached statistical significance. In any case, both therapies led to significant reductions in borderline symptoms, although no significant difference was found between them, which is in line with previous studies assessing the relative efficacy and specificity of similar staggered psychotherapeutic interventions in BPD. For example, a recent study found that a 12-week short-term stepped treatment can be as effective as a 24-month longer-term treatment for most BPD patients [39]. Even 1 month of weekly contact has been demonstrated to reduce demand in terms of hospital units by BPD patients with acute suicidal risk [40].

These results show that, after IT and after supportive therapy, the symptoms of BPD were reduced; the magnitude was comparable to those found by other well-established psychotherapies using the BSL-23 as the primary outcome [41]. In addition, IT was associated with greater satisfaction compared to non-specific SI at the end of treatment. However, this difference was not maintained over time, as satisfaction at follow-up was similar for both groups. The differential effects of IT visual images (immediate) and SI verbal words (delayed) on information processing speed may partly explain the initial difference between groups. The similar therapeutic effects for both interventions may be related to progressive equalization over time. These results are partially in line with studies showing that specific group therapies give better results [42] for patients with BPD traits, although it is necessary to carry out more extensive trials with IT to assess its specificity.

Several RCTs have not shown significant differences when comparing two different psychological interventions in BPD [43–46], whereas others have shown differences; however, the majority of these used small samples or have a high risk of bias [47, 48]. Generally, evidence on the differential effectiveness of psychological interventions is very poor. Considering the large overlap between supportive and Iconic Therapy, with shared focus on psychoeducation and similar ways of planning and structuring their programs (see Table 2 of the study protocol) [31], the medium effect size of the difference (d = 0.43) between the two therapy groups in BSL- 23 mean scores at 12 months is remarkable. More studies are needed to determine which factors are responsible for the improvement of outcomes.

Regarding applicability and implementation in the real world, these results should be aligned with the results of other studies indicating alternative staggered treatment for BPD [12], because the accessibility of long treatments such as behavioral dialectic therapy or mentalization-based treatment is limited due to the large amount of resources needed and the lack of experienced therapists. This type of brief therapy would adapt especially well to a young population with acute symptoms [49]. Furthermore, in certain patients, mainly in the early stages of the disorder, long-term treatment may not be necessary, as several studies suggest [41, 50–52]. These results are linked to the body of evidence suggesting that structured preventive strategies in the early stages of the disorder may be an adequate alternative [53].

One of the main limitations of this study is that the sample size was not of the required size due to unexpected difficulties in the recruitment process; thus, the sample was small and underpowered for detecting small effect sizes. A second limitation is that, due to recruitment problems, the inclusion and exclusion criteria needed to be modified by extending the age range and by not using an instrument that was in the study protocol. Furthermore, structured questionnaires were used for the selection of participants, not structured clinical interviews; however, the questionnaires show adequate psychometric properties. Finally, individuals may have different learning styles in terms of approaching and processing information (i.e. visual, auditory or tactile), which may hinder the understanding and assimilation of IT for some of participants. Despite these limitations, the trial shows some strengths, such as the low percentage of drop-outs in the follow-up (20%) and the large percentage of participants who completed at least 80% of the sessions (57.5%). Another strength is that the trial was conducted in a real world scenario where clinical staff were included along with non-experienced therapists, thus the study had high external validity.

## Conclusions

In this preliminary study, a significant reduction in BPD symptoms was found in both treatment groups (IT and SI). The difference between the effectiveness of the two groups was not statistically significant, as usually occurs when comparing different BPD psychotherapies. However, an effect size was found for reducing the symptoms of BPD and maladjustment in daily life at the 12-month follow-up, which may indicate some superiority of IT. Moreover, participants in the IT group showed greater satisfaction with therapy than those who received SI, after the 10-month treatment, and there was a lower percentage of non-suicidal self-injuries, at the 12-month follow-up. In any case, future studies with larger samples and comparisons with established treatments for borderline personality disorder are necessary to confirm or deny these results.

## Data Availability

All methods were performed in accordance with the relevant guidelines and regulations. The datasets used during the current study will be available under reasonable request to the corresponding author.

## Abbreviations

APA: American Psychology Association
BPD: Borderline personality disorder
BSL-23: Borderline Personality Disorder List
CEPER-III-BPD: Exploratory Questionnaire of Personality-III-BPD
CEQ: Satisfaction with therapy
C-SSRS: Columbia Suicide History Form
DBT: Dialectical Behaviour Therapy
DSM-5: Diagnostic and Statistical Manual of Mental Disorders, Fifth Edition
GEE: Generalized estimating equations
GLM: Generalized linear models
IG: Maladjustment Scale
MBT: Mentalization-based Therapy
NICE: National Institute for Health and Care Excellence
SCID-II: Clinical Interview for DSM-IV Axis II Disorders
SFT: Schema-focused Cognitive Therapy
STEPPS: Systems Training for Emotional Predictability and Problem Solving
TAU: Treatment as usual
TFP: Transference-focused Therapy
